# Collective punishment is more effective than collective reward for promoting cooperation

**DOI:** 10.1038/srep17752

**Published:** 2015-12-04

**Authors:** Lei Gao, Zhen Wang, Riccardo Pansini, Yao-Tang Li, Rui-Wu Wang

**Affiliations:** 1 Center for Ecological and Environmental Sciences, Northwestern Polytechnical University, Xi’an, 710072, P.R. China; 2 School of Mathematics and Information Science, Beifang University of Nationalities, Yinchuan, 750021, P.R. China; 3 School of Mathematics and Statistics, Yunnan University, Kunming, Yunnan, 650091, P.R. China; 4 State Key Laboratory of Genetic Resources and Evolution, Kunming Institute of Zoology, Chinese Academy of Science, Kunming, Yunnan, 650223, P.R. China; 5 Interdisciplinary Graduate School of Engineering Sciences, Kyushu University, Fukuoka, 816-8580, Japan

## Abstract

Collective punishment and reward are usually regarded as two potential mechanisms to explain the evolution of cooperation. Both scenarios, however, seem problematic to understand cooperative behavior, because they can raise the second-order free-rider problem and many organisms are not able to discriminate less cooperating individuals. Even though they have been proved to increase cooperation, there has been a debate about which one being more effective. To address this issue, we resort to the N-player evolutionary snowdrift game (NESG), where a collective punishment/reward mechanism is added by allowing some players to display punishment/reward towards all remaining players. By means of numerous simulations and analyses, we find that collective punishment is more effective in promoting cooperation for a relatively high initial frequency of cooperation or for a relatively small group. When the intensity of punishment exceeds a certain threshold, a stable state of full cooperation emerges for both small and large groups. In contrast, such state does not appear for large groups playing a NESG with reward mechanism. In the case of mutualistic interactions, finally, our results show the new payoff with collective punishment/reward can lead to the coexistence of cooperators and defectors when discrimination between these two is not possible.

Behavior we define as cooperation is widely observed in nature from interacting genes to multi-cellular organisms, yet explaining the evolution of cooperation has been a conundrum for sociologists, economists and biologists alike^1^,^2^,^3^. To date, the classical theories of kin selection^4^, group selection^5^,^6^, reciprocal altruism^1^,^7^, indirect reciprocity^8^,^9^ and spatial reciprocity^10^ have been proposed to explain the evolution and the maintenance of cooperation. Among them, kin selection and reciprocal altruism argue that organisms who endorse cooperative strategies receive a fitness advantage if the partners share common genes or exchange beneficial acts. These adaptations are considered as active processes, because the partners are voluntarily induced to cooperate. Therefore, the evolutionarily stable strategy of cooperation becomes gradually fixed in the populations^11^,^12^.

Although reciprocal altruism and kin selection are both accepted as important dynamics to explain the evolution of cooperation, they encounter significant problems in relation to cheating. They in fact give little hints on why and how some individuals at times change their strategy into a non-cooperative one to the extent of becoming cheaters^13^,^14^,^15^. In these instances, cooperation turns into competition causing conflicts amongst the players who fight for the limited resources available^16^,^17^,^18^,^19^,^20^,^21^. To overcome these problems, recent empirical observations suggest that cooperation may have evolved through an enforcement mechanism^22^,^23^,^24^,^25^,^26^,^27^. This mechanism might have evolved through sanctioning or punishing less cooperative or non-cooperative behaviors. These behaviors happen to be exerted by mutualistic hosts and by queens, kings, or workers of eusocial animals; on the opposite side of the spectrum, these ‘game changer’ players can reward the cooperative actors^23^,^26^,^27^,^28^.

Cognition was generally believed to be an important ability to discern cooperators and non-cooperators^29^. This is not possible, however, when organisms with simple nervous systems cannot give proof of implementing such discerning abilities mediated by their consciousness^29^. This is likely to be the case of many symbiotic interactions^30^,^31^,^32^. The free riding symbionts or subordinates, in addition, may find it difficult to know when and how intense the punishment or the reward is going to be^33^,^34^,^35^. Yet importantly, some defectors may deliberately try to cheat naive partners by mimicking cooperators, thereby avoiding punishment^36^,^37^. We conclude here that being able to discriminate cheaters is often not possible in nature. It is of interest, therefore, to understand how cooperation systems could have evolved exactly when individuals do not show signs of using any discrimination process.

In the present paper, under the theoretical framework of the multiplayer snowdrift game, we develop a collective punishment model in the absence of a discrimination mechanism. For promoting cooperation, our results show that such collective policing mechanism is more effective than a reward mechanism. Moreover, we show how collective punishment can allow the maintenance of mutualism when discriminating between cooperators and defectors is not possible. Our results describe and justify these claims.

## Model and Results

### Background

In the prisoner’s dilemma (PD) game^38^, an individual needs to decide to either cooperate or defect when interacting with another player. If both players cooperate, each get a payoff of *R* points, compared with the lower *P* points in case both decide to defect. If they choose different behaviors, the player who defects receives the higher score of *T* points, whereas the player who cooperates gets the lower score of *S*. With *T* > *R* > *P* > *S* and 2*R* > *S* + *T*, the dilemma becomes apparent: regardless of the opponent’s choice, an individual is better off defecting in a game consisting of a single round. This reasoning should lead the anonymous players to consistently choose mutual defection even though mutual cooperation carries a greater reward.

The dilemma of the PD game can be eased in the modification brought forward by the snowdrift game (SG). This game assumes that cooperation yields a benefit *b* allocated to both players. The costs *c*, instead, are divided between the cooperators^39^. The payoff matrix of the SG shows that each player can receive a reward, *R* = *b* − *c*/2, from the mutual cooperation (Table 1). A defector, instead, obtains a payoff of *T* = *b* from playing against a second cooperator, whereas the latter obtains a payoff of *S* = *b* − *c* > 0 (*b* > *c* > 0). In the last case of mutual defection, each player receives the payoff of *P* = 0. Here, *T* > *R* > *S* > *P* is different from the ranking *T* > *R* > *P* > *S* of the PD game with 2*R* > *T* + *S*. From the payoff matrix we see that, the strategy best to choose depends on the opposing player’s strategy, resulting in a mixed evolutionary stable state. The frequency of cooperation becomes, then, 1 − *c*/(2*b* − *c*).

The above pairwise game is usually not appropriate in biology and human societies, because the individuals do not live in simple dyadic relationships but they are most often found in multiplayer interactions. As a metaphor, the multiplayer snowdrift game is often employed to investigate how the cooperation evolves between many-to-many interactions^30^,^40^,^41^,^42^. Examples of group foraging, territorial defense, predator defense, and sucrose metabolism are all instances of multiplayer snowdrift games^43^,^44^. Similarly to existing literature^45^, we use Π_*C*_(*k*) and Π_*D*_(*k*) to denote the payoffs of a cooperator and a defector:


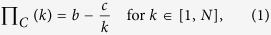



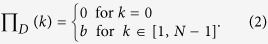


where *k* is the number of cooperators in interacting groups and *N* represents the group size. It follows that the extent of cooperation decreases with increasing cost-to-benefit ratio *c*/*b* and the number *N* of interacting players in the population^45^.

### N-player evolutionary snowdrift game with collective punishment in a single species (intra-specific cooperation)

Inspecting the two payoffs (1) and (2), some benefit can be produced as long as at least one cooperator is found in their group. This model, however, ignores the fact that the interacting players bear some additional costs when fewer players share the cooperation work. In cooperative hunting species (for a general carnivores’ account, see ref. 46, including the most notable behaviour of lions^47^), obtaining prays takes more efforts and time when fewer cooperators engage in hunting (as recently found in the Malagasy fosa^48^). As a result, the amount of food intake decreases. This decrease can be regarded as an additional cost for the hunting lions. Another example of collective cooperation, this time in humans, is project management. We are sometime faced with complex projects to carry out by a team of persons. Project management is a topic of study we have developed to specifically avoid all the possible drawbacks. Hence, a good project manager will put in place strategies to avoid that some employees defect from their duties and that the fewer cooperators incur in additional work (in project designing, cf.^49^).

Additional costs are considered in our model as collective punishment for each participant. This collective punishment originates usually from an external pool of resources or through compulsory contributions made by all participants^50^,^51^. By definition, collective punishment is imposed on all players either because there is no way to detect the behavior of individual players (or its effects), or because the differences between cooperators and non-cooperators are too small to be detected. Under these assumptions, we can obtain the payoff value for a cooperator and a defector, respectively:


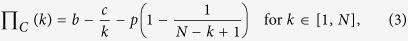



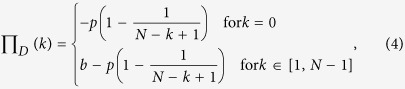


where 
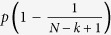
 is the additional punishment, which is a marginal function of the number of cooperators *k* and group size *N*. The parameter *p* represents the intensity of punishment. Each participant gets a maximum punishment of 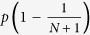, when no individual chooses to cooperate in the group.

Considering a well-mixed population, the frequency of the cooperative players is given by *x*(*t*). On the other hand, the fitness of a cooperator and a defector are given by *f*_*C*_ and *f*_*D*_, respectively. Via random sampling the groups^52^,^53^, we obtain the following average fitness of a cooperator and a defector:


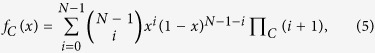



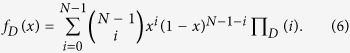


Thus, the average fitness of the player is


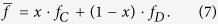


The replicator dynamics assumes that the change in frequency of a strategy is proportional to the difference between the fitness of that strategy and the average fitness of the species 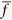
54,55,56. Thus, the time evolution of the frequency of cooperation is


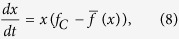


Substituting equation (7) into equation (8), the dynamics of *x*(*t*) becomes


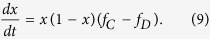


It is not difficult to see that the model can be solved analytically for small groups. For large groups, instead, we must resort to numerical simulation to study the existence and stability of the inner equilibrium points, because the dynamic equation (9), with *N* + 1 powers, becomes too complex.

From equation (9), we obtain two boundary equilibrium points *x*_0_ = 0 and *x*_1_ = 1. By analyzing the stability of equation (9), we find that *x*_0_ = 0 is unstable and that *x*_1_ = 1 keeps stable if *p*/*b* > 2*c*/(*Nb*)(for further reasoning on this point, see Supporting Informationsir>). In addition, we find that the inner equilibrium point 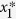 is stable and the other inner equilibrium point 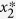 is unstable (see Fig. 3(d,e)). This means that, starting from different initial values of *x*(*t*), the system is expected to evolve in time, and the solution trajectories should converge into the equilibrium points *x*_1_ = 1 or 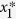 (see Fig. 3(d,e)).

### N-player evolutionary snowdrift game with collective punishment or reward between two species (mutualistic cooperation)

All above games have been used to analyze intra-specific cooperation with the addition of collective punishment. This was usually implemented in single group of individuals, with no further analyses for inter-specific mutualisms (or, more generally, interactions between groups). We know very well, though, that symbiotic relationships are ubiquitous in nature. Examples of mutualistic cooperation have been found between several species (as reviewed by^17^, and with examples coming from e.g.^57^,^58^,^59^). In this sense, evolutionary snowdrift game has been widely employed to study such inter-specific relationships^40^,^60^,^61^. While most of these achievements exploring cooperation and conflict in symbioses presuppose that the cooperative species do not produce any reward or punishment. This assumption is undoubtedly invalid for many mutualistic interactions. For example, in most well-documented mutualisms such as figs and fig wasps, legumes and nitrogen-fixing bacteria, and cleaner fish and its client, the dominant species or hosts can set the rules of the game between mutualistic species. Consistent with this principle, hosts of these mutualisms were shown to sanction non-cooperative actors or reward cooperative actors to maintain the mutualistic interaction. In addition, the punishment or reward of hosts in above mutualisms is usually imposed on all individuals in symbionts either because there is no ability for these hosts to detect the behaviour of individual partners (or its effects) or because the differences between cooperative and non-cooperative individuals are too small to be detected (as in the legume–rhizobium mutualism^32^). It is therefore of interest to explore the effects of the punishment and reward mechanisms in the evolution of mutualisms.

**Figure 1:**
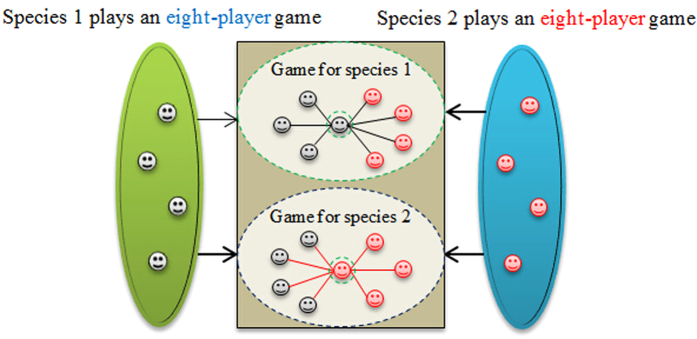
Illustration of interactions between species 1 and 2 in mutualisms. We assume that the number of members of each species is four. Species 1 and 2 both play in an eight-player game. In this first game, we choose one player of species 1 (the grey faces) to interact with remaining players species 1 and four players of species 2 (the red faces). Species 2 plays an eight-player game too. In this second game, one player of species 2 joins and interacts with remaining players species 2 and all the players of species 1.

In this model, we assume that individuals of species 1 and 2 are selected at random from an infinitely large, well-mixed population, and form an interacting group of size *N*. The members of the two species engage in multiplayer game, and the illustration of interactions is shown in Fig. 1. Here we will study a special case of above interactions with one player in species 1 and *N*-1 player in species 2, because in realistic systems such as figs and fig wasps, legumes and nitrogen-fixing bacteria, cleaner fish and its client, or ants and larvae, a single dominant individual (e.g., fig, legume, big fish and larvae) is tended to by multiple subordinate individuals (e.g., fig wasps, nitrogen-fixing bacteria, cleaner fish and ants).

Now we extend multiplayer snowdrift games with collective punishment, which can easily reflect real-world situations in mutualisms. We assume that dominant species or hosts (species 1) will pay some costs to impose a fine of punishment 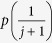 towards each participant of its partner (species 2) because of the speculative behavior. The costs of punishment originate usually from compulsory contributions made by all participants in dominant species 1. As a result, each individual of species 1 will pay a cost of punishment, 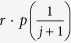 (*r* ≤ 1). The term 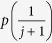 gives an estimate of the size of the punishment as a marginal function of the number of cooperative individuals *j* in subordinate specie 2. The parameter *p* represents the intensity of the punishment, and the parameter *r* represents the proportion of loss of the punisher (dominant species). In addition, we assume the mutualistic benefit is provided only if the number of cooperators in the species 1 and 2 both exceed one (i.e., *i* ≥ 1 and *j* ≥ 1), or otherwise, no benefit can be produced and each cooperators will pay a maximum cost 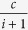 or 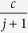. Following these assumptions, the payoff values for a cooperative individual and a defector of species 1 and 2 can be written as


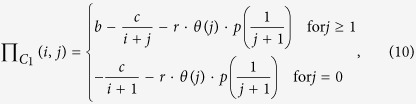



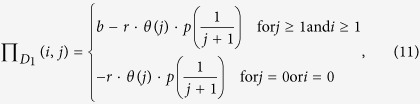


and


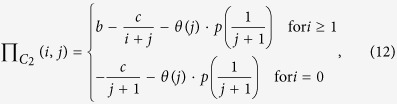



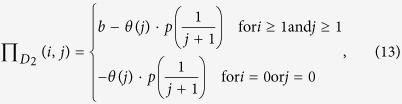


where 
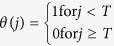
 is the Heaviside function, which means that punishment will work only if the number of cooperators *j* (0 ≤ *j* ≤ *N* − 1) in species 2 is below a threshold value *T* (1 ≤ *T* ≤ *N* − 1); the parameters *b* and *c* are all positive real numbers; the number of cooperators *i* + *j* in the interacting groups is a real number between 0 and *N*. Similarly, we can obtain a NESG model with a collective reward mechanism (see Supporting Information). Here, we only analyze the effect of collective punishment to keep in mind that the effect of collective reward can be analyzed in an analogous way (for an analysis details of collective reward, see Supporting Information).

The cooperation frequency in species 1 and species 2 is denoted by *x*(*t*) and *y*(*t*), respectively. The fitness functions of a cooperative individual of species 1 and species 2 are 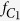 and 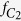, respectively. Random sampling the groups^52^,^53^, we obtain the following average fitness of cooperators and defectors of species 1 and 2, respectively,


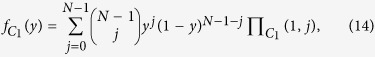



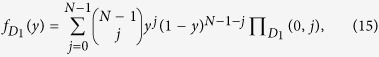


and


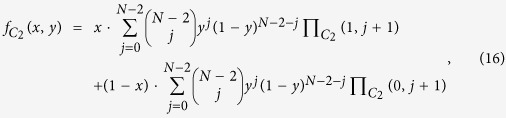



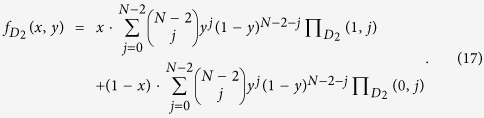


Thus, the average fitness of the players of species 1 and 2 is


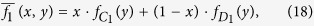


and


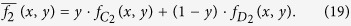


Adding the rule of the replicator dynamics as assumption, it follows that:


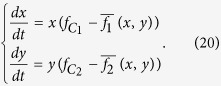


From equation (20), we can obtain four boundary equilibrium points: *E*_1_(0, 0), *E*_2_(0, 1), *E*_3_(1, 0) and *E*_4_(1, 1), where both cells of vector (*x*, *y*) are respectively the frequency of cooperation in species 1 and 2. For large groups, we must resort to numerical simulation to study the existence and stability of the inner equilibrium points, because the dynamic equation (20) with *N* + 1 powers is remarkably complex. We detect then an inner equilibrium point *E*_5_(x^*^, y^*^) and two boundary equilibrium points *E*_6_(1, y^**^) and *E*_7_(0, y^***^) (with 0 < x^*^, y^*^, y^**^, y^***^ < 1), for a different punishment-to-benefit ratio *p*/*b* (see Figs 4 and 5). Through analysis and simulation (see the electronic supplementary material, and Figs 4 and 5), we find that the equilibrium point *E*_1_(0, 0) is sink (stable) at [1 − *θ*(1)/2]*p* < *c*/2. The equilibrium points *E*_2_(0, 1), *E*_3_(1, 0) and *E*_5_(x^*^, y^*^) are sources (unstable). The equilibrium point *E*_4_(1, 1) is sink (stable) at *c*/*N* < [*θ*(*N* − 2)/(*N* − 1) − *θ*(*N* − 1)/*N*]*p*, and the equilibrium point *E*_6_(1, y^**^) is sink (stable). In addition, the boundary equilibrium point *E*_7_(0, y^***^) is source (unstable).

## Simulations and Discussions

Enforcement by punishment or sanctioning has been suggested as one of the most important dynamics in the evolution of cooperation^33^,^62^,^63^,^64^. Widespread forms of a posteriori cooperation strategy in single species are those of sanctioning or punishing less cooperative or non-cooperative behavior, while rewarding cooperation^65^,^66^,^67^. Examples of these strategies adopted by mutualistic partners, are in particular found between legumes and rhizobia^25^,^68^, yuccas and yucca moths^35^,^69^, ants and acacias^70^, and fig trees and fig wasps^26^,^31^,^71^. Hosts or dominant partners (e.g., legumes, yucca plants, and fig trees) may often have difficulties to distinguish between the cooperators and non-cooperators. However, we know that they can still respond to the collective action of their partners (e.g., yuccas^23^, fig trees^26^, and rhizobia^32^). In these cases, instead of using individual punishment or reward, we show that the behaviors of collective punishment and collective reward are more appropriate to illustrate the concerted interactions between individuals.

Here, we will discuss three points. (1) The effectiveness of the collective punishment and collective reward mechanisms. Although most of previous studies have concluded that these a posteriori strategies to punish and to reward can promote cooperation, their true positive effects have been challenged by recent studies^66^,^72^,^73^,^74^. (2) Which is more effective to enforce cooperation between the collective punishment and the collective reward^62^,^65^,^75^,^76^,^77^,^78^,^79^? (3) How cooperation systems could evolve in the absence of a discrimination mechanism? This has already been discussed by Archetti & Scheuring^30^, who argue that if two groups of individuals trade goods (benefits) that are non-linear, their mutualistic interaction is maintained. The interaction can therefore be maintained by the exchange of these public goods, even when it is not possible to punish defectors.

### Collective punishment effect within species (intra-specific cooperation)

**Figure 2:**
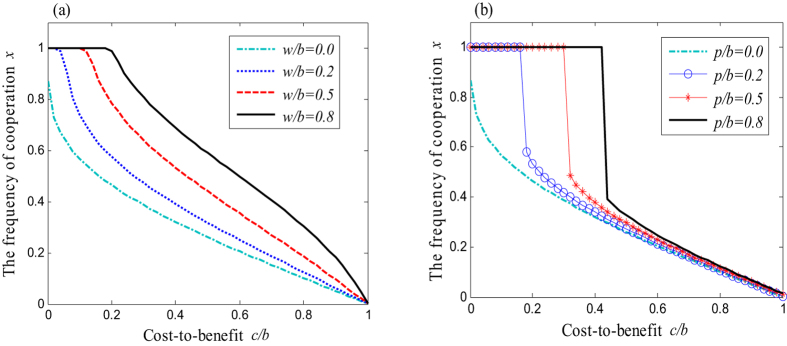
Cooperative frequency *x* in dependence on the cost-to-benefit ratio *c*/*b* for different values of *p*/*b* and *w*/*b*. (**a**) Reward mechanism can effectively promote cooperation, and the frequency of cooperation will reach 1 for a small cost-to-benefit ratio *c*/*b*^80^. (**b**) However, the punishment enables cooperation to reach completely dominated state for a relative large cost-to-benefit ratio *c*/*b*. The competing group size here is *N* = 5, and the initial frequency of the cooperation for all simulation is fixed at 0.5.

**Figure 3:**
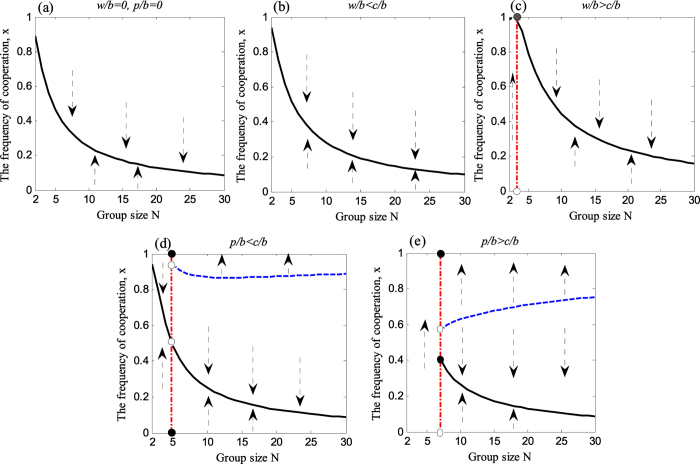
Stationary fraction of cooperation. It is shown that the stable equilibrium 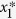 (thick lines) and unstable equilibrium 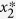 (dotted lines) as a function of group size *N* for different values of reward-to-benefit ratio *w*/*b* and punishment-to-benefit ratio *p*/*b*. Between these equilibria, the fraction of cooperator either increases or decreases as a result of the evolutionary dynamics, which is indicated by arrows here. (**a**) Even without rewarding and punishing, a stable equilibrium exists for a small cost-to-benefit ratio, *c*/*b*. However, the fraction of cooperators drops with the increase of cost-to-benefit ratio, *c*/*b*. (**b**,**c**) show when the reward ratio increases, a stable state of full cooperation emerges for small group size^80^. (**d**) Considering the punishment in this game (namely, instead of the reward mechanism), besides the interior equilibrium, the boundary equilibrium is also stable. A stable state of full cooperation (ALLC state) emerges for large group size when *p*/*b* < *c*/*b*, (**e**) while ALLC state emerges for any group size when *p*/*b* > *c*/*b*. Parameters: *c*/*b* = 0.2; (**a**) *w*/*b* = 0, *p*/*b* = 0; (**b**) *w*/*b* = 0.1, *p*/*b* = 0; (**c**) *w*/*b* = 0.5, *p*/*b* = 0; (**d**) *w*/*b* = 0, *p*/*b* = 0.1; (**e**) *w*/*b* = 0, *p*/*b* = 0.5.

It can be seen from Fig. 2(b) that the frequency of cooperation is promoted with the increment of punishment intensity *p*. In particular, when the intensity of punishment exceeds a certain threshold of *p*/*b* > 2*c*/(*Nb*) (see Figs 2(b) and 3(d,e) for a relative small cost-to-benefit ratio), cooperation can even reach the completely dominated state. Similarly, with the collective reward mechanism (for more details also refer to the Supporting Information), when the intensity of the rewards exceeds the certain threshold, *w*/*b* > (*N* − 1)*c*/*b*, the frequency of cooperation can raise to a very high level (*x* = 1) as well (see Figs 2(a) and 3(c))^80^. Combining with these observations that, it is clear that collective punishment and reward is effective in promoting cooperation for a relative high initial frequency of cooperation or for a relative small group.

In addition, another interesting finding is that collective punishment seems more effective than collective reward (because collective reward can only support complete cooperation at small cost-to-benefit ratio *c*/*b*). For small groups, both the collective reward and the collective punishment can effectively promote cooperation; especially when the reward-to-benefit *w*/*b* and the punishment-to-benefit *p*/*b* exceed the ratio of cost-to-benefit *c*/*b* (see Fig. 3(c,e), in which the arrows point to the different levels of cooperation). For large groups, instead, we find that collective punishment can result in the emergence of a stable state of full cooperation (*x* = 1) (Fig. 3(d,e)), while such a harmonious ALLC state does not emerge when the collective reward mechanism is carried out (Fig. 3(b,c)). These observations imply that the effectiveness of collective rewards for promoting full cooperation declines in large groups while collective punishment remains effective. In fact, in large societies of social animals or insects, instances of punishment are more commonly practiced in respect to reward. In relation to this, we cite a very representative example. To prevent exploitation, social insects have evolved several policing methods. The best known is the one of “worker policing”, whereby the workers destroy the eggs laid by other workers. This phenomenon was first documented in the honeybee^81^. Since then, it has been discovered in more than 15 species of bees, wasps, and ants^82^. In addition, our results are consistent with similar ones pointing at the dilemma arising when choosing the “carrot versus the stick”. Likewise, such studies also concluded that punishing is more effective than rewarding^65^,^83^,^84^,^85^.

Finally, we explore the reasons of why punishing is more effective than rewarding. Humans and other animals show, in the short run, amplified awareness and respond promptly with a drive towards self-regulation. In this specific case, this drive is exerted with a more circumstantial adaptation to an environment occupied by willing cooperators. In most species, we do not share the argument that the punished targets experience negative effects that in the long run give rise to a counterproductive behavior^84^,^86^,^87^. The positive effects, such as those that provide a feeling of satisfaction and contentment seem to wear off more quickly. In such situations, the organisms may be motivated to cooperate to a lesser degree (as also discussed by^87^). Finally, yet importantly for cognitively advanced organisms, the reception of negative information seems to psychology cause greater emotional impact than the reception of equivalent positive information^86^. As a result, organisms that are able to process and deal with negative cues appropriately (including non-human primates^88^) will show a fitness advantage in the social environment they live in.

### Collective punishment effect between species (mutualistic cooperation)

**Figure 4:**
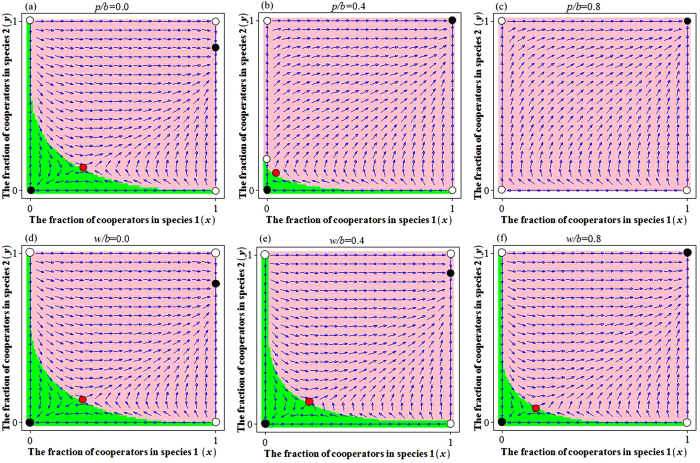
The cooperative behaviors in N-player evolutionary snowdrift game, depending on the values of punishment-to-benefit ratio *p*/*b* or the reward-to-benefit ratio *w*/*b*. The ‘green region’ and the ‘pink region’ are the basins of attraction of *E*_1_(0, 0) and *E*_6_(1, y^**^) (or *E*_4_(1, 1)), respectively. (**a**) Without punishment mechanism, the game has two ESSs: *E*_1_(0, 0) and *E*_6_(1, y^**^). The solution trajectories converge to the point *E*_6_(1, y^**^) if the initial strategy frequencies of each species fall within the ‘pink region’, whereas they converge to the point *E*_1_(0, 0) if the initial strategy frequencies fall within the ‘green region’. (**b**,**c**) However, the ‘green region’ vanishes as well as a global state of full cooperation emerges for a large punishment-to-benefit ratio *p*/*b*. Considering the collective reward mechanism, there are two ESSs: *E*_1_(0, 0) and *E*_6_(1, y^**^) (or *E*_4_(1, 1)) in the systems for any reward-to-benefit ratio *w*/*b* (as in (**d**–**f**)). Parameters: *N* = 3, *b* = 1, *c* = 0.5, *r* = 0.5, *T* = 2; (**a**) *p*/*b* = 0.0; (**b**) *p*/*b* = 0.4; (**c**) *p*/*b* = 0.8; (**d**) *w*/*b* = 0.0; (**e**) *w*/*b* = 0.4; (**f**) *w*/*b* = 0.8.

**Figure 5:**
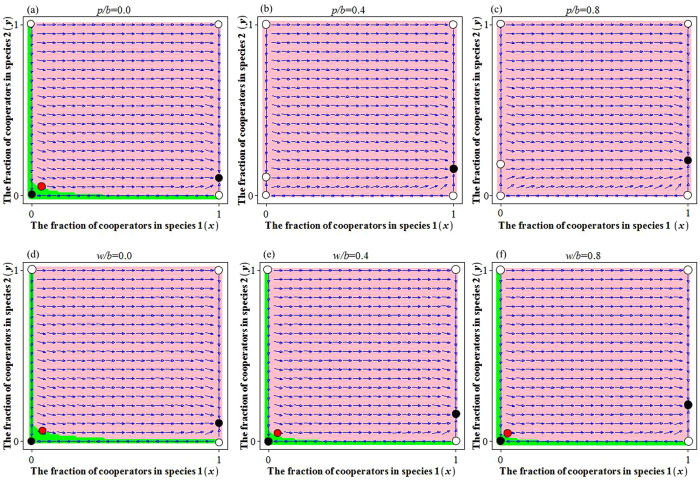
The cooperative behaviors in N-player evolutionary snowdrift game, depending on the values of punishment-to-benefit ratio *p*/*b* or the reward-to-benefit ratio *w*/*b* in large groups. The stable equilibrium point *E*_6_(1, y^**^) may always exist, no matter how much severe the punishment-to-benefit ratio *p*/*b* (as in (**a**–**c**)), or the reward-to-benefit ratio *w*/*b* (as in (**d**–**f**)). In addition, *E*_1_(0, 0) is always stable for collective reward mechanism (as in (**d**–**f**)). Parameters are *N* = 20, *b* = 1, *c* = 0.5, *r* = 0.5, *T* = 2; (**a**) *p*/*b* = 0.0; (**b**) *p*/*b* = 0.4; (**c**) *p*/*b* = 0.8; (**d**) *w*/*b* = 0.0; (**e**)*w*/*b* = 0.4; (**f**) *w*/*b* = 0.8.

In the classical snowdrift game between two species, there is a mixed Nash equilibrium which is unstable (it is in fact a non-ESS). This means that the species involved are often better off defecting than cooperating^40^. However, this situation can be eased by adding the new payoff with collective punishment\reward element into the game. By doing so, the mixed Nash equilibrium becomes stable, and the frequency of cooperation is promoted by increasing the punishment-to-benefit ratio *p*/*b* or by increasing the reward-to-benefit ratio *w*/*b* (see the ‘pink regions’ of Figs 4 and 5). Besides, a stable state of full cooperation (ALLC state) emerges when *c*/*N* < [*θ*(*N* − 2)/(*N* − 1) − *θ*(*N* − 1)/*N*]*p*. Specifically, it can be seen from Figs 4 and 5 that the system has two stable equilibrium points *E*_1_(0, 0) and *E*_6_(1, y^**^). We point out that the ESS *E*_6_(1, y^**^) is a boundary equilibrium, which implies that the players in species 1 always cooperate and the players in species 2 cooperate with a probability y^**^ (see the ‘pink regions’ of Figs 4 and 5), and the cooperation level y^**^ is promoted by increasing of the punishment-to-benefit ratio *p*/*b* (see Figs 4(a–c) and 5(a–c)), or by increasing of the reward-to-benefit ratio *w*/*b* (see Figs 4(d–f) and 5(d–f)). In addition, the equilibrium point *E*_1_(0, 0) becomes unstable for a relative large punishment-to-benefit ratio *p*/*b* (see Figs 4(b) and 5(b)), but it is stable for any size of the reward-to-benefit ratio *w*/*b* (see Figs 4(d–f) and 5(d–f)).

Our results are consistent with some empirical evidence showing that punishment and reward solve the conflicts between the actors of mutualisms. For example, in the obligate inter-specific cooperation between figs and fig wasps, the fig trees may not distinguish between the pollinating wasps and non-pollinating, parasitic wasps. As a consequence, the fig trees respond to the collective action of all wasps by selectively aborting syconia (the enlarged receptacles with multiple ovaries) or decreasing the offspring/development ratio^26^,^89^, therefore showing sanctioning. In addition, cooperative pollinator wasps would be predicted to increase in numbers with the additional immigrating individuals. Such immigrants should be encouraged to move away because of the high rewards offered by the fig plants, resulting in high offspring developmental rates. As a result, the stability of mutualisms can be maintained, thereby avoiding the tragedy of the commons.

## Conclusion

To explore how cooperative systems evolve in the absence of cognition, we have proposed a collective punishment mechanism, and incorporate it into the multiplayer snowdrift game. Moreover, we also compared the effectiveness of collective punishment and collective reward for promoting cooperation. Our model demonstrates, for a relatively high initial frequency of cooperation or for a relatively small group, that collective punishment is more effective than collective reward for promoting cooperation. When the punishment-to-benefit ratio *p*/*b* exceeds a certain threshold of 2*c*/(*Nb*), the cooperative behavior is overall enhanced. It is interesting that a global stable state of full cooperation (ALLC state) emerges for small groups, and a local stable ALLC state emerges for large groups. In contrast, when considering the classical NESG or the NESG with reward mechanism for large group size *N*, such a harmonious ALLC state does not appear. These results are consistent with other studies pointing at the “carrot versus the stick” dilemma. Accordingly, punishment becomes more effective than reward in sustaining public cooperation^65^,^83^,^84^,^85^.

In a game scenario attended by two different interacting species, our results show that the players in species 1 always cooperate and the players in species 2 cooperate with a certain probability, which depends on the initial cooperation frequency combined with the punishment intensity. That is to say, the stability of mutualisms can be maintained by the use of a new payoff setup with collective punishment/reward. At the same time, the cooperation is promoted by increasing the punishment-to-benefit ratio *p*/*b*, and the ALLC stable states will gradually spread together with the increment of the punishment intensity for small groups.

## Additional Information

**How to cite this article**: Gao, L. *et al.* Collective punishment is more effective than collective reward for promoting cooperation. *Sci. Rep.* **5**, 17752; doi: 10.1038/srep17752 (2015).

## Supplementary Material

Supplementary Information

## Figures and Tables

**Table 1:** Payoff matrix of the Snowdrift Game.

		*Player* 2	
		*C*	*D*
*Player* 1	*C*	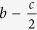	*b* − *c*
	*D*	*b*	0
