# Supplementary material for: Collective punishment is more effective than collective reward for promoting cooperation
Source: Sci Rep. 2015 Dec 4;5:17752. doi: 10.1038/srep17752 (PMC4669458; doi:10.1038/srep17752)
Supplement: Supplementary Information [file srep17752-s1.pdf]

Supplementary Information

Collective punishment is more effective than collective reward for promoting cooperation

Lei Gao[1,2,3*], ZhenWang[1,4,*,#], Riccardo Pansini[2], Yao-Tang Li[3], Rui-Wu Wang[1,2,#]

[1] Center for Ecological and Environmental Sciences, Northwestern Polytechnical University, Xi'an, 710072, China.

[2] State Key Laboratory of Genetic Resources and Evolution, Kunming Institute of Zoology, Chinese Academy of Science, Kunming, Yunnan, 650223, P.R. China.

[3] School of Mathematics and Statistics, Yunnan University, Kunming, Yunnan, 650091, P.R. China.

[4] Interdisciplinary Graduate School of Engineering Sciences, Kyushu University, Fukuoka, 816-8580, Japan

* These authors contributed equally to this work.

# zhenwang0@gmail.com (ZW); ruiwukiz@hotmail.com(RWW).

Dynamics and Evolutionary Stability of the Snowdrift Game

Appendix A

Considering the cooperative behavior in a single species, the evolution time of $x(t)$ of the players are governed by

$$\frac{dx}{dt} = x \cdot (1-x) \cdot (f_C - f_D), \tag{s1}$$

we can obtain two boundary equilibrium points, $x_0 = 0$ and $x_1 = 1$, and the inner

equilibrium point x^*, which satisfies the equation $f_C(x^*) - f_D(x^*) = 0$. Since $(f_C - f_D)|_{x=0} = b - c + p/[N \cdot (N+1)]$ and $(f_C - f_D)|_{x=1} = \frac{p}{2} - \frac{c}{N}$, we get that the boundary equilibrium point $x_0 = 0$ is unstable and the boundary equilibrium point $x_1 = 1$ is stable if $p/b > 2c/(bN)$. For the inner equilibriums point x^*, x^* is stable if $(\partial(f_C - f_D)/\partial x)|_{x=x^*} < 0$. Here, we usually consider large group sizes. In this case we must resort to simulations, because the dynamic equation (s1) with $N+1$ power becomes very complex. Through simulations, we find the system has two inner equilibrium points, x_1^* and x_2^*. The inner equilibrium point x_1^* is stable and the other inner equilibrium point x_2^* is unstable (see fig.2c and 2d in main text).

Appendix B

A collective reward model within a single species was proposed by Ji et al. (2010). The payoffs of the cooperator and the defector are given by, respectively

$$\Pi_C(k) = b - \frac{c}{k} + w \cdot \left(1 - \frac{1}{k}\right) \quad \text{for} \quad k \in [1, N], \tag{s2}$$

and

$$\Pi_D(k) = \begin{cases} 0 & \text{for } k = 0 \\ b + w \cdot \left(1 - \frac{1}{k}\right) & \text{for } k \in [1, N-1] \end{cases} . \tag{s3}$$

$w(1 - \frac{1}{k})$ is the additional reward, when the work is completed sooner as a result of more cooperators sharing the work, all involved players could enjoy the fruit of the joint effort earlier and thus they should all receive an additional reward, and w represents the intensity of reward. In this game, cooperation can be enhanced by

increasing the reward-to-benefit ratio w/b. For small group size, a stable state with full of cooperation ($x=1$) should emerge when the intensity of reward exceeds a certain threshold [1].

We consider a game played between species in mutualisms, and assume that dominant species or hosts (species 1) will pay some costs so as to bring a reward $w\cdot\left(1-\frac{1}{i+j}\right)$ to each individual of its partners (species 2) because of the cooperative behavior. The costs of reward originate usually from compulsory contributions made by all participants in dominant species 1. As a result, we assume that each individual of species 1 will gain less reward, $r\cdot w\cdot\left(1-\frac{1}{i+j}\right)$ ($r\le 1$). The term $w\cdot\left(1-\frac{1}{i+j}\right)$ gives an estimate of the size of the reward as a function of the number of cooperators $i+j$ in the interacting group. The parameter w represents the intensity of the reward, and the parameter r represents the discount of reward for the dominant species 1. In addition, we assume the mutualistic benefit is provided only if the number of cooperators in the species 1 and 2 both exceed one (i.e., $i\ge 1$ and $j\ge 1$), or otherwise, none benefit can be produced and each cooperators will pay a maximum cost $\frac{c}{i+1}$ or $\frac{c}{j+1}$. Under these assumptions, the N-player evolutionary snowdrift game model with collective reward mechanism between species can be given by

$$\Pi_{C_1}(i,j)=\begin{cases} b-\frac{c}{i+j}+r\cdot\pi(j)\cdot w\left(1-\frac{1}{i+j}\right) & \text{for } j\ge 1 \\ -\frac{c}{i+1} & \text{for } j=0 \end{cases} \tag{s4}$$

$$\prod_{D_1}(i,j)=\begin{cases} b+r\cdot\pi(j)\cdot w\left(1-\dfrac{1}{i+j}\right) & \text{for } j\geq 1 \text{ and } i\geq 1 \\ 0 \quad \text{for } j=0 \text{ or } i=0 \end{cases}, \tag{s5}$$

$$\prod_{C_2}(i,j)=\begin{cases} b-\dfrac{c}{i+j}+\pi(j)\cdot w\left(1-\dfrac{1}{i+j}\right) & \text{for } i\geq 1 \\ -\dfrac{c}{j+1} \quad \text{for } i=0 \end{cases}, \tag{s6}$$

$$\prod_{D_2}(i,j)=\begin{cases} b+\pi(j)\cdot w\left(1-\dfrac{1}{i+j}\right) & \text{for } i\geq 1 \text{ and } j\geq 1 \\ 0 \quad \text{for } i=0 \text{ or } j=0 \end{cases}, \tag{s7}$$

where $\prod_{C_m}(i,j)$ and $\prod_{D_m}(i,j)$ denote the payoffs of a cooperator and a defector of species m (with m=1, 2); $\pi(j)=\begin{cases} 0 & \text{for } j<T \\ 1 & \text{for } j\geq T \end{cases}$ is the Heaviside function, which means that reward will work only if the number of cooperators *j* ($0\leq j\leq N-1$) in species 2 is greater than or equal to a threshold value T ($1\leq T\leq N-1$); the parameters *b* and *c* are all positive real numbers, and the number of cooperators *i*+*j* in the interacting groups is a real number between 0 and *N*.

Appendix C

The evolution time of $x(t)$ and $y(t)$ of the two species are governed by the following replicator dynamic equations:

$$\begin{cases} \dfrac{dx}{dt}=x\left(f_{C_1}(y)-\overline{f_1}(x,y)\right) \\ \dfrac{dy}{dt}=y\left(f_{C_2}(x)-\overline{f_2}(x,y)\right) \end{cases}. \tag{s8}$$

i.e.

$$\begin{cases} \frac{dx}{dt} = x(1-x)\left(f_{C_1} - f_{D_1}\right) \equiv F(x,y) \\ \frac{dy}{dt} = y(1-y)\left(f_{C_2} - f_{D_2}\right) \equiv Q(x,y) \end{cases} . \quad \text{(s9)}$$

From equation (s9), we can obtain four boundary equilibrium points denoted as $E_1(0,0)$, $E_2(1,0)$, $E_3(0,1)$ and $E_4(1,1)$. In addition, using simulations, we can obtain one inner equilibrium point $E_5\left(x^*, y^*\right)$ and two boundary equilibrium points $E_6\left(1, y^{**}\right)$ and $E_7\left(0, y^{***}\right)$ where $0 < x^*, y^*, y^{**}, y^{***} < 1$. The elements of vector (x, y) are respectively the frequencies of the cooperators of species 1 and 2. It is however difficult to obtain analytical solutions of x^*, y^*, y^{**} and y^{***} for large groups, because the dynamic equation (s9) with $N+1$ powers is remarkably complex.

The stability of the nonlinear system can be obtained from the evolutionary stability analysis of linearization[2]. Thus, to analyze the evolutionary stability of replicator dynamics (s9) with N+1 power, we should first obtain the linearization of the dynamics at every equilibrium point. The linearization of the replicator dynamics (s9) at an equilibrium point $(\bar{x}, \bar{y})$ becomes

$$\begin{pmatrix} dx/dt \\ dy/dt \end{pmatrix} = \begin{pmatrix} \partial F(x,y)/\partial x & \partial F(x,y)/\partial y \\ \partial Q(x,y)/\partial x & \partial Q(x,y)/\partial y \end{pmatrix}\Bigg|_{(x,y)=(\bar{x},\bar{y})} \cdot \begin{pmatrix} x \\ y \end{pmatrix}, \quad \text{(s10)}$$

where

$$\partial F(x,y)/\partial x = (1-2x)\left(f_{C_1} - f_{D_1}\right), \quad \text{(s11)}$$

$$\partial F(x,y)/\partial y = x(1-x)\cdot\frac{\partial\left(f_{C_1}-f_{D_1}\right)}{\partial y}, \tag{s12}$$

$$\partial Q(x,y)/\partial x = y(1-y)\cdot\frac{\partial\left(f_{C_2}-f_{D_2}\right)}{\partial x}, \tag{s13}$$

and

$$\partial Q(x,y)/\partial y = (1-2y)\left(f_{C_2}-f_{D_2}\right)+y(1-y)\cdot\frac{\partial\left(f_{C_2}-f_{D_2}\right)}{\partial y}. \tag{s14}$$

By analyzing the property of the matrices' eigenvalues, produced by the linearization of the replicator dynamics (s9) at the equilibrium points, we find that the equilibrium point $E_1(0,0)$ is sink (stable) at $\left[1-\theta(1)/2\right]p < c/2$; the equilibrium points $E_2(0,1)$, $E_3(1,0)$ and $E_5\left(x^*,y^*\right)$ are sources (unstable); the equilibrium point $E_4(1,1)$ is sink (stable) at $c/N < \left[\theta(N-2)/(N-1)-\theta(N-1)/N\right]p$.

References

1 Ji, M., Xu, C., Zheng, D.-F. & Hui, P. Enhanced cooperation and harmonious population in an evolutionary N-person snowdrift game. *Physica A* **389**, 1071-1076 (2010).

2 Hirsch, M. W., Smale, S. & Devaney, R. L. *Differential equations, dynamical systems and an introduction to chaos* (Academic Press, 2004).
